# Long-Term In Vivo Biological Performance of PLLA–b–PEG/HA Filler

**DOI:** 10.3390/jfb17090460

**Published:** 2026-09-08

**Authors:** Shujiang Zhang, Tong He, Shuhan Wang, Lixin Yuan, Hongjiang Liu, Ruizhi Li, Kun Zhang, Shiwei Wang, Chen Lai

**Affiliations:** 1 The First Affiliated Hospital of Guangzhou Medical University, Guangzhou 510182, China; drzhangsj@gzhmu.edu.cn; 2 Beijing Key Laboratory of Aging Regulation and Translational Innovation in Skin and Soft Tissue, Beijing 100022, China; hetong@imeik.com (T.H.); liruizhi@imeik.com (R.L.); zhangkun@imeik.com (K.Z.); 3 Beijing Engineering Lab of Neo-Biodegradable Materials, Beijing 100022, China; 4 Shenzhen Institute for Drug Control (Shenzhen Testing Center of Medical Devices), Shenzhen 518057, China; wangshuhan@szidc.org.cn (S.W.); yuanlixin@szidc.org.cn (L.Y.); liuhongjiang@szidc.org.cn (H.L.); 5 Biomedical Engineering Center, Peking University Shenzhen Institute, Shenzhen 518057, China

**Keywords:** PLLA–b–PEG, hyaluronic acid, biostimulatory filler, neocollagenesis, neoelastogenesis, long-term biocompatibility, tissue remodeling

## Abstract

**Objective:** This study aimed to evaluate the long-term degradation behavior, biostimulatory effects, and biocompatibility of a novel poly-L-lactic acid-block-polyethylene glycol/hyaluronic acid (PLLA–b–PEG/HA) composite filler for soft tissue augmentation. **Methods:** PLLA–b–PEG/HA microsphere properties were characterized via scanning electron microscopy (SEM), X-ray diffraction (XRD), Fourier-transform infrared spectroscopy (FTIR), nuclear magnetic resonance hydrogen spectroscopy (^1^H NMR), thermogravimetry (TG) and differential scanning calorimetry (DSC). A 104-week in vivo rabbit model was established to systematically observe filler degradation and tissue responses. Ultrasound monitoring, histological staining, ELISA and RT-PCR were performed to assess volumetric changes, inflammatory reactions and collagen synthesis-related signaling. **Results:** Physicochemical property tests demonstrated that PLLA–b–PEG retains the fundamental physicochemical properties of pristine PLLA while exhibiting enhanced hydrophilicity. B-ultrasound demonstrated a presented uniform in vivo distribution without displacement or diffusion over time, confirming steady and predictable degradation. SEM verified progressive morphological degradation and porous evolution of the microspheres. The filler induced a mild, balanced inflammatory microenvironment with early expression of both pro-inflammatory (IL-12, TNF-α) and anti-inflammatory (IL-4) cytokines, which resolved gradually over time. Sustained TGF-β upregulation persisted throughout the 104-week observation period, driving continuous neocollagenesis and prominent neoelastogenesis, thereby achieving favorable and long-term tissue remodeling with excellent biocompatibility. **Conclusions:** The PLLA–b–PEG/HA composite filler exhibits controllable degradation properties and homeostatic regulatory effects, along with outstanding long-term biosafety and tissue integration capacity. As an ideal biostimulatory filler for soft tissue augmentation, it can effectively facilitate the regeneration of high-quality functional extracellular matrix rich in collagen fibers and elastic fibers, and holds promising clinical prospects for natural and long-lasting soft tissue filling applications.

## 1. Introduction

Novel facial fillers based on PLLA–b–PEG microspheres have attracted growing research and clinical attention, owing to the unique combination of the intrinsic collagen-stimulating properties of PLLA and the superior biocompatibility conferred by PEG modification. Several studies have validated the application prospects of PLLA–b–PEG-based fillers, which are commonly compounded with hyaluronic acid (HA) to achieve immediate volumetric filling and sustained long-term biostimulation for facial contour reconstruction, volume restoration, and skin rejuvenation [1,2,3]. Such composite fillers are capable of promoting neocollagenesis, ameliorating skin laxity, and lowering the incidence of adverse events such as nodule formation compared with conventional pure PLLA fillers, exhibiting prominent clinical advantages [4,5,6].

The interactions between microspheres and host tissue differ markedly across distinct implantation anatomical layers, including the superficial dermis, subcutaneous fat layer and deep supraperiosteal regions. Clarification of such site-specific biological interactions enables clinicians to achieve predictable clinical application of PLLA–b–PEG fillers. Deep injection is suitable for structural volumization and facial lifting, while superficial subdermal injection with diluted fillers contributes to skin tightening and quality improvement [7]. Clarifying site-specific tissue-material interactions is essential to standardize clinical injection protocols, including needle/cannula selection, injection depth, single-site dosage, and postoperative care, thereby achieving predictable and individualized therapeutic outcomes.

Traditional histological evaluation relies on invasive biopsy sampling, which is traumatic, scar-forming, and only provides static cross-sectional information at a single time point. In contrast, non-invasive imaging technologies, including high-frequency ultrasound (HFUS) [8], magnetic resonance imaging (MRI) [9], and optical coherence tomography (OCT) [10], enable continuous dynamic in vivo monitoring of the same implantation site. In particular, high-frequency ultrasound can precisely quantify injection depth, newly formed collagen thickness, and acoustic impedance differences among native tissue, residual filler, and neogenetic fibrotic tissue, allowing for quantitative assessment of the filler’s biostimulatory efficacy. It provides objective morphological and volumetric indicators for evaluating filler degradation kinetics and biostimulatory efficacy, serving as an ideal auxiliary tool for long-term in vivo filler assessment.

In addition to material mass loss, dynamic changes in the microscopic morphology of degradable microspheres are critical for evaluating filler biocompatibility. The in vivo hydrolytic degradation of PLLA–b–PEG microspheres is driven by the cleavage of ester bonds within the polymer backbone, and this process is regulated by multiple factors. First, the content of PEG blocks is a key determinant: their inherent hydrophilicity enhances water penetration into the polymer matrix, which significantly accelerates the hydrolysis rate of ester bonds. Second, the crystallinity of PLLA segments acts as a physical barrier, such that higher crystallinity corresponds to a slower hydrolysis rate. In addition, environmental pH exerts a dual regulatory effect: alkaline environments directly accelerate hydrolysis, and the carboxylic acid end groups generated during the degradation process can form an acidic microenvironment inside the microspheres, producing a self-catalytic effect that further promotes degradation. Along with the ongoing hydrolysis, microspheres exhibit reduced particle size and an increased surface-area-to-volume ratio [11,12]. Non-uniform hydrolysis may transform smooth spherical microspheres into porous structures or irregular fragments. Notably, irregular and sharp micro-debris can induce excessive mechanical stimulation to surrounding tissues and further activate inflammatory cascades, potentially driving pathological foreign body granuloma formation and compromising therapeutic safety [13].

Although existing short-term and medium-term results have confirmed the favorable performance of PLLA–b–PEG fillers [14,15], comprehensive long-term observational data exceeding 1–2 years are still lacking, which limits the systematic understanding of their sustained volumetric correction efficacy, persistent collagen-stimulating effects, and long-term biosafety profiles. While collagen stimulation has been observed, the specific cellular and molecular mechanisms triggered by PLLA–b–PEG microspheres, including distinct immune responses and differential tissue interactions across different anatomical layers, remain to be further elucidated. Long-term follow-up studies of more than one year are therefore urgently needed.

This study aimed to characterize the inherent properties of PLLA–b–PEG/HA filler through laboratory examinations, and evaluate its biocompatibility, long-term safety and efficacy via in vivo animal experiments at different implantation layers combined with non-invasive ultrasonic detection. It also explored its long-term degradation pattern, regulatory effects on inflammatory responses at implantation sites, modulatory effects on collagen and elastic fiber regeneration, as well as differential tissue responses in different implantation layers, so as to lay solid experimental and theoretical foundations for its clinical application in facial contouring.

## 2. Materials and Methods

### 2.1. Verification of Physical and Chemical Properties

#### 2.1.1. Materials

PLLA–b–PEG microspheres and PLLA powder were supplied by the Beijing Engineering Laboratory of Novel Biodegradable Materials. For PLLA–b–PEG microspheres, the weight-average molecular weight (Mw) was 1.251 × 10^5^ with a polydispersity index (PDI) of 1.601; 95% of the microspheres had a particle size ranging from 20 to 45 μm, with an average particle size of 32 μm. For PLLA powder, the Mw was 1.131 × 10^5^ with a PDI of 1.812; 95% of the powder particles had a size ranging from 20 to 80 μm, with an average particle size of 57 μm. The cross-linked sodium hyaluronate used in the filler formulation was sourced from Kewpie (Tokyo, Japan), with a weight-average molecular weight (Mw) of 9.564 × 10^5^ and a PDI of 1.302. The injectable suspension used for the experiments was a cross-linked sodium hyaluronate gel incorporated with PLLA–b–PEG microspheres, developed by Imeik Technology Development Co., Ltd. (Beijing, China). Its formulation consisted of 180 mg/mL PLLA–b–PEG microspheres, 17 mg/mL sodium hyaluronate, and 3 mg/mL lidocaine hydrochloride. This product has obtained approval from the National Medical Products Administration (NMPA) of China.

#### 2.1.2. Physicochemical Characterization of Materials

The macroscopic and microscopic morphology of samples was observed using a field emission scanning electron microscope (SEM), including freeze-dried composite gel and pure microspheres.

X-ray diffraction (XRD) was performed to detect microsphere samples and acquire data on crystallinity and unit cell parameters.

Fourier-transform infrared spectroscopy (FTIR) was used to analyze functional groups and characteristic spectra of materials within the scanning range of 400–4000 cm^−1^.

Nuclear magnetic resonance hydrogen spectroscopy (^1^H NMR) was used to analyze functional groups and characteristic spectra of the materials within the scanning range of 0–10 ppm, with deuterated chloroform (CDCl_3_) as the solvent.

Thermogravimetry (TG) and differential scanning calorimetry (DSC) were conducted to investigate the thermal properties of microspheres. Thermal curves were plotted to analyze thermal history, including crystallization temperature, glass transition temperature and melting point.

### 2.2. In Vivo Tissue Implantation Analysis

#### 2.2.1. Animals

Thirty 4-month-old male New Zealand rabbits (body weight: 2.2–2.5 kg) were obtained from Beijing Longan Experimental Animal Breeding Center (Beijing, China). The animals were housed under controlled environmental conditions (temperature: 23 ± 3 °C; relative humidity: 40–70%; 12 h light/dark cycle) and provided with standard laboratory chow and purified water ad libitum. Following a 1-week acclimation period, the rabbits were randomly assigned to five experimental groups (6 rabbits per group), corresponding to the predefined observation time points: 4, 26, 52, 78, and 104 weeks. All experimental procedures were reviewed and approved by the Institutional Animal Care and Use Committee (IACUC No. YXKT2023L012, approval date: 18 May 2023) of Beijing YongXin Kangtai Technology Development Co., Ltd. (Beijing, China).

#### 2.2.2. Experimental Procedure

Anesthesia was induced by intramuscular injection of a xylazine compound (0.1 mL/kg) combined with tiletamine-zolazepam (0.1 mL/kg), and maintained with isoflurane (Qingdao Orbiepharm Co., Ltd., Qingdao, China) in a sterile operating room. The hair over the implantation sites on the rabbits’ parietal skull and dorsal region was shaved, followed by sequential disinfection of the skin with alcohol and iodophor. A 27-gauge needle was used for the subcutaneous implantation of 0.5 mL of the filler at each designated site: a single site at the midpoint of the parietal skull, and six sites (3 cm apart bilaterally) along the paravertebral region of the back.

#### 2.2.3. Ultrasonic Testing

Ultrasound examinations were performed on the dorsal skin of the rabbits in the 104-week group (the same six rabbits) using the MyLabSix ultrasound system (Esaote, Genoa, Italy) at 4, 26, 52, 78, and 104 weeks post-injection. The epidermal layer presented a linear hyperechoic signal, and the implant area exhibited a well-defined, high-resolution grayscale image with uniformly distributed, homogeneous echoes. After image acquisition, the longitudinal diameter, transverse diameter, and vertical height of the implant sites were precisely measured using electronic calipers.

#### 2.2.4. Tissue Morphological Observation

At 4, 26, 52, 78, and 104 weeks post-injection, biopsy specimens containing the epidermal layer, subcutaneous tissue, and underlying parietal bone were harvested from the implantation sites under general anesthesia, with meticulous care taken to preserve the native tissue architecture. Control tissue samples were obtained from non-implanted anatomical regions using an identical harvesting protocol. The excised tissues were fixed in 10% neutral-buffered formalin, and osseous components were decalcified prior to standard paraffin embedding. Full-thickness 4-μm tissue sections were cut along the sagittal plane, with the implantation site as the central axis.

Sections were stained with hematoxylin and eosin (H&E), Masson’s trichrome, Alcian blue, Picrosirius red, Herovici’s Histology and Movat’s pentachrome stains. Picrosirius red-stained sections were imaged using a polarizing microscope (Ci–S, Nikon Corporation, Tokyo, Japan) for the differentiation of type I and type III collagen, whereas all other stained sections were examined under a standard light microscope (BX63, Olympus Corporation, Tokyo, Japan). Quantitative analysis of collagen and hyaluronic acid (HA) content in the implantation region was performed using Image-Pro Plus 6.0 software (Media Cybernetics, Bethesda, MD, USA).

#### 2.2.5. Scanning Electron Microscopy

At 4, 26, 52, 78, and 104 weeks post-injection, central subcutaneous implantation site tissues were harvested from rabbit backs under general anesthesia, and each sample was bisected. One half was immediately placed in electron microscopy (EM)-specific fixative (2.5% glutaraldehyde) for morphological preservation, while the other was refrigerated for subsequent PLLA–b–PEG microsphere elution. Refrigerated samples underwent sectioning, grinding, and enzymatic hydrolysis, followed by microsphere elution via suction filtration. EM-fixed tissues and eluted PLLA–b–PEG microspheres were separately attached to conductive adhesive tapes, sputter-coated at 10 mA for 45 s, and morphologically observed using a scanning electron microscope (SEM; TESCAN MIRA LMS, Brno, Czech Republic) at 2 kV acceleration voltage. Images were acquired with the SE2 secondary electron detector.

#### 2.2.6. Rt-Pcr

At 4, 26, 52, 78, and 104 weeks post-implantation, tissues from the subcutaneous implantation sites were collected and immediately frozen in liquid nitrogen for total RNA extraction. Total RNA was isolated using RNAiso Plus reagent in accordance with the manufacturer’s protocol. Subsequently, complementary DNA (cDNA) was synthesized via reverse transcription, followed by reverse transcription-polymerase chain reaction (RT-PCR) to detect the expression levels of the target genes: Wnt oncogene analog 10 (Wnt10), Catenin beta (β-catenin), Alpha-Smooth Muscle Actin (α-SMA), Collagen I, and Collagen III.

#### 2.2.7. Elisa

Biopsy samples collected at 4, 26, 52, 78, and 104 weeks post PLLA–b–PEG/HA implantation were flash-frozen in liquid nitrogen and homogenized. Enzyme-linked immunosorbent assay (ELISA) was performed according to the manufacturer’s instructions to determine the expression levels of interleukin 12 (IL-12), tumor necrosis factor-alpha (TNF-α), transforming growth factor-beta (TGF-β), and interleukin 4 (IL-4)—molecules commonly used to assess implant-associated inflammatory responses and collagen production.

#### 2.2.8. Statistical Analysis

Statistical analyses and graphical visualization were performed using GraphPad Prism 10 (GraphPad Software, Inc., San Diego, CA, USA). In vivo experimental data were expressed as the mean ± standard error of the mean (SEM). For all in vivo quantitative assays, one implantation site was sampled per animal, and the statistical unit “n” represents the number of independent animals (n = 6 per time point). Intergroup differences among multiple groups were assessed using one-way analysis of variance (ANOVA), and unpaired Student’s *t*-tests were used for pairwise comparisons. A *p*-value of less than 0.05 (*p* < 0.05) was considered statistically significant.

## 3. Results

### 3.1. In Vitro Physicochemical Characterization Results

**Figure 1:**
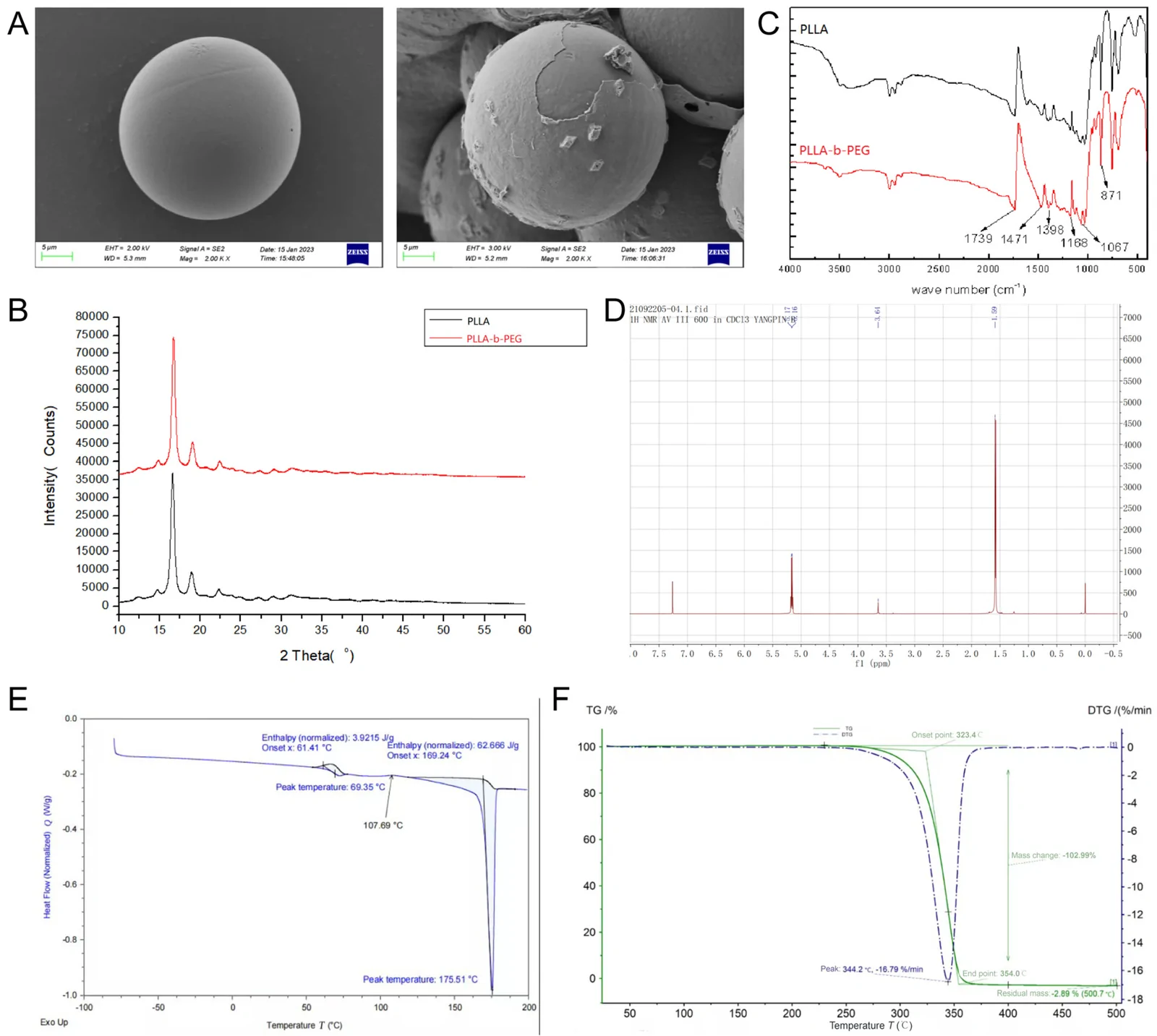
**In vitro physicochemical characterization of PLLA–b–PEG/HA filler.** (**A**) Scanning electron microscopy (SEM) images: left panel, pure PLLA–b–PEG microspheres; right panel, freeze-dried PLLA–b–PEG/HA filler (magnification: 2000×); (**B**) X-ray diffraction (XRD) patterns of pristine PLLA and PLLA–b–PEG; (**C**) Fourier-transform infrared (FTIR) spectra of pristine PLLA and PLLA–b–PEG; (**D**) ^1^H NMR characterization verified the successful synthesis of the PLLA–b–PEG block copolymer; (**E**) Thermogravimetric (TG) and derivative thermogravimetric (DTG) curves of PLLA–b–PEG; (**F**) Differential scanning calorimetry (DSC) curve of PLLA–b–PEG.

SEM images of PLLA–b–PEG microspheres and freeze-dried microsphere/HA composite gel are presented in Figure 1A (magnification: 2000×). The microspheres exhibited regular spherical morphology with smooth surfaces and particle sizes ranging from 20 to 45 μm. After incorporation into HA gel and freeze-drying, the microspheres were uniformly wrapped by lyophilized sodium hyaluronate, retaining intact spherical shape and consistent particle size. This homogeneous encapsulation is attributed to both the improved hydrophilicity of the PEG-modified microspheres and specific intermolecular interactions. Hydrogen bonding serves as the dominant physicochemical mechanism: the abundant ether bonds (-O-) on the surface PEG segments of PLLA–b–PEG microspheres form multiple hydrogen bonds with the hydroxyl groups (-OH) distributed along HA chains. FTIR evidence from previous studies confirms this interaction, as the C–O–C stretching vibration peak of PEG shifts from 1110.82 cm^−1^ to 1079.96 cm^−1^ after mixing with HA, a typical red shift caused by hydrogen bond formation. Quantitative studies further show that the PEG-HA complex has an apparent binding constant of approximately 45,000 ± 8000 M^−1^, with about 3.3 ± 0.1 ethylene glycol units bound per HA disaccharide unit [16]. This hydrogen-bond-mediated interaction also induces conformational contraction of HA chains, which helps form a tighter and more stable composite structure between microspheres and the HA gel matrix. Surface wrinkles observed on microspheres were derived from the dried gel matrix, and no fragmentation was detected.

XRD patterns of PLLA and PLLA–b–PEG powders are shown in Figure 1B. PLLA displayed typical α-crystal diffraction peaks at 16.7° (110/200), 19.1° (203) and 22.4° (211), with no miscellaneous peaks. The XRD profile of PLLA–b–PEG was nearly identical to that of pure PLLA in terms of peak positions and intensities, indicating that the introduction of PEG segments did not alter the crystalline form of PLLA. Notably, no characteristic diffraction peaks of PEG (typically around 19.2° and 23.3°) were detected in the copolymer sample. This absence of PEG crystalline signals can be attributed to two factors. First, the PEG segment in the copolymer has a relatively low molecular weight (1000 Da), which endows it with inherently weaker crystallization ability compared with high-molecular-weight PEG. Second, in this diblock copolymer system, PLLA segments with much higher molecular weight (1.251 × 10^5^ Da) crystallize preferentially during cooling, and the covalently linked PEG chains are spatially confined within the interlamellar or interfibrillar amorphous regions [17]. This nanoscale confinement severely restricts the chain folding and packing of PEG segments, thereby inhibiting the formation of long-range ordered PEG crystals.

FTIR spectra showed consistent characteristic absorption peaks for PLLA and PLLA–b–PEG: 871 cm^−1^ (C–C stretching), 1067 cm^−1^ (asymmetric C–O stretching), 1168 cm^−1^ (C–O–C stretching), 1398 cm^−1^ (symmetric –CH_3_ deformation), 1471 cm^−1^ (–CH_3_ bending) and 1739 cm^−1^ (C=O stretching). No clearly distinguishable new peaks were observed in the copolymer spectrum (Figure 1C). This lack of apparent spectral difference is explained by three factors. First, the PEG segment has a molecular weight of only 1000 Da, accounting for approximately 0.77% of the total copolymer mass, so its infrared signal is weak and largely masked by the strong background of PLLA. Second, the characteristic C–O–C stretching peak of PEG at ~1100 cm^−1^ severely overlaps with the C–O–C stretching peak of the PLLA backbone at ~1090 cm^−1^, preventing clear resolution. Third, as indicated by the XRD results, PEG segments exist in an amorphous state within the copolymer, which broadens their absorption peaks and further reduces detectability. Given that conventional FTIR cannot independently confirm the presence of PEG blocks, we further verified the copolymer structure via ^1^H NMR. The characteristic peak at δ = 3.64 ppm, corresponding to the methylene protons of PEG segments, confirmed the successful synthesis of the PLLA–b–PEG block copolymer (Figure 1D).

The TG curve of PLLA–b–PEG powder is shown in Figure 1E, and its thermal degradation proceeded in three overlapping stages. The first stage occurs from approximately 200 °C to 300 °C, with only 5–10% total mass loss. This stage corresponds to chain-end depolymerization of PLLA segments: terminal hydroxyl and carboxyl groups with lower thermal stability trigger “back-biting” reactions, releasing small amounts of water, lactide precursors and low-molecular-weight oligomers. The second stage (320 °C to 370 °C, with a maximum degradation rate at 344 °C) is the main decomposition stage, accounting for over 70% of total mass loss. It arises from random scission of ester bonds along the PLLA backbone via intramolecular cis-elimination and transesterification reactions, generating large amounts of lactide and volatile small molecules. The third stage is attributed to thermal degradation of PEG segments via free-radical cleavage of C–O–C ether bonds, which typically occurs at 370–450 °C for pure PEG. In this copolymer system, however, acidic degradation products released during PLLA decomposition exert a catalytic effect, shifting PEG degradation to lower temperatures and causing it to largely overlap with the PLLA main decomposition stage. This explains why no independent mass loss plateau is observed for the third stage, and the sample is almost completely pyrolyzed by approximately 400 °C.

The DSC curve of PLLA–b–PEG is shown in Figure 1F, and three main thermal transitions can be identified. First, a weak endothermic peak appears at 61.41–69.35 °C (peak temperature: 69.35 °C, enthalpy: 3.9215 J/g), which is assigned to the melting of confined PEG crystalline domains. The elevated melting temperature relative to pure PEG-1000 and the extremely low melting enthalpy are attributed to the short PEG chain length (1000 Da) and strong spatial confinement imposed by the high-molecular-weight PLLA matrix; the calculated crystallinity of PEG is approximately 2%. Notably, the glass transition of PLLA (typically 55–60 °C for high-molecular-weight PLLA) overlaps with this PEG melting peak, so it cannot be distinguished as an independent step transition. Second, a broad cold crystallization peak (Tc) is observed at 107.69 °C. This transition corresponds to the rearrangement and ordering of amorphous PLLA chain segments upon heating above Tg, indicating that the PLLA phase did not fully crystallize during sample preparation. Third, a sharp and intense endothermic peak at 175.51 °C (enthalpy: 62.666 J/g) corresponds to the complete melting of PLLA crystals. The narrow peak width indicates good crystallite perfection and relatively uniform molecular weight distribution of PLLA segments. Based on the theoretical melting enthalpy of 100% crystalline PLLA (~93 J/g), the crystallinity of the PLLA phase is calculated to be approximately 67.4%.

### 3.2. In Vivo Ultrasound Manifestation and Volume Change Trend of the PLLA–b–PEG/HA Filler

**Figure 2:**
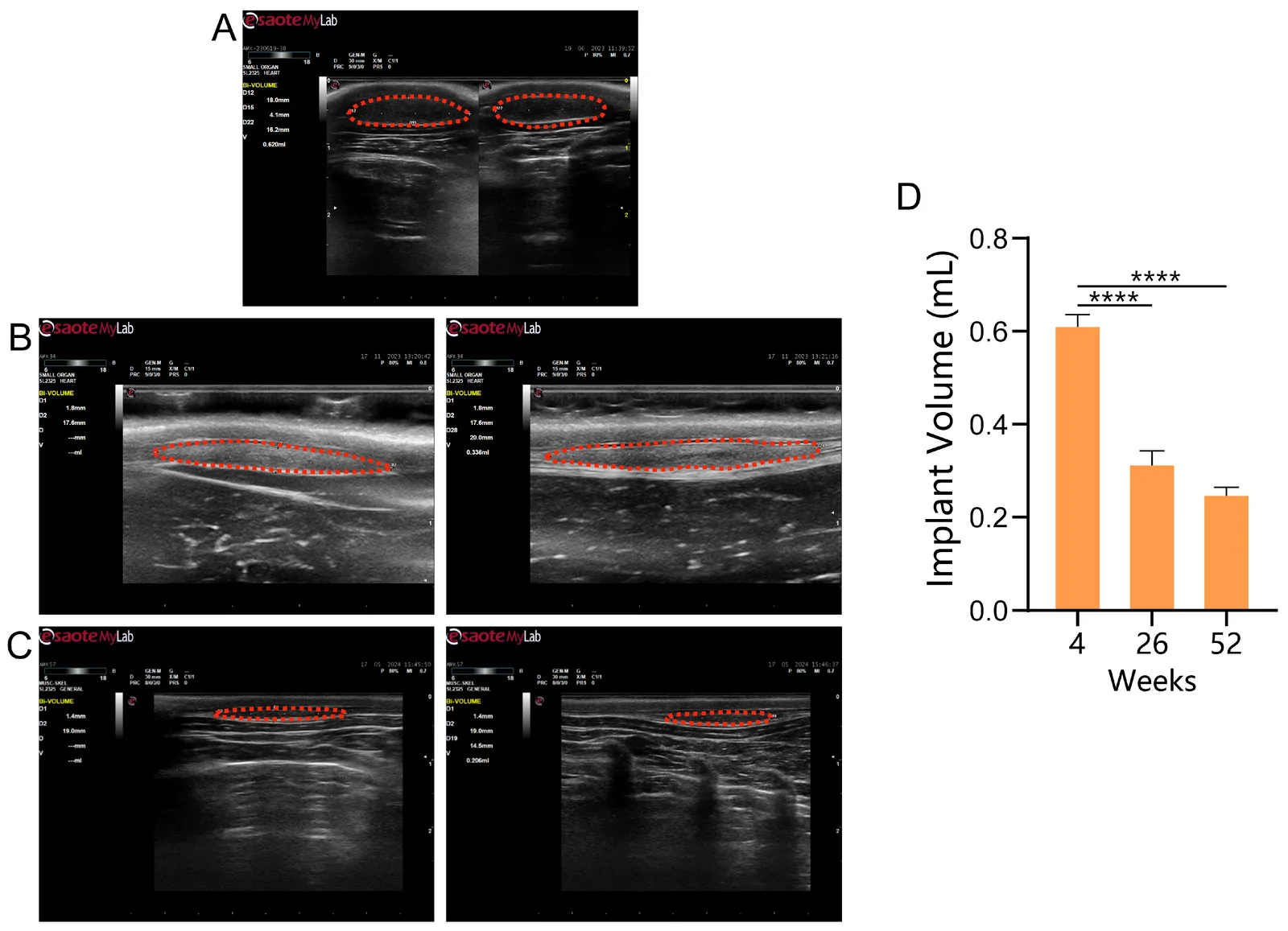
**Implant volume of material detected by B-ultrasound.** (**A**) Implant volume at 4 weeks after injection (dotted red line). (**B**) Implant volume at 26 weeks after injection (dotted red line). (**C**) Implant volume at 52 weeks after injection (dotted red line). (**D**) Implant volume statistics at different times after injection. **** *p* < 0.0001, n =  6. Data are presented as mean  ±  SEM. SEM, Standard error of the mean.

Ultrasound was used to examine the implantation area at the dorsal skin of rabbits. Implants were visible at 4, 26, and 52 weeks post-implantation, presenting an overall homogeneous echo pattern with clear outlines and regular shapes throughout the entire observation period. No material diffusion around the implant, implant displacement, or other abnormal morphological manifestations were observed. The implant volume decreased gradually with the extension of implantation time. The inherent hypoechoic feature of the filler began to be gradually replaced by hyperechoic signals from fibrous connective tissue proliferation starting at 26 weeks post-implantation. At 78 and 104 week after implantation of the test samples, no obvious implant-specific echo signal was detected in the subcutaneous implantation area (Figure 2).

### 3.3. Tissue Responses After In Vivo Implantation at Different Sites

Compared with the control group, tissue reactions induced by the PLLA–b–PEG/HA filler in vivo were observed at all time points after implantation in the rabbit periosteum and subcutaneous. At 4 and 26 weeks post-implantation, with the gradual degradation of hyaluronic acid, the PLLA–b–PEG microspheres were gradually exposed from the matrix; notably, more macrophages were distributed around the exposed microspheres, while the microspheres still embedded in the matrix were not surrounded by a large number of inflammatory cells. At 4, 26, and 52 weeks post-implantation, the degree of inflammatory reaction in the periosteum and subcutaneous implantation areas gradually increased, reaching a peak at 52 weeks, and then began to decrease at 78 weeks. At this time (78 weeks), no intact PLLA–b–PEG microspheres were visible in the implantation areas—this time point, which is of particular concern due to microsphere fragmentation, showed macrophages phagocytizing microsphere fragments, but no obvious sharp increase in inflammatory reaction was observed. By 104 weeks post-implantation, the tissue reaction had basically returned to normal (Figure S1).

This inflammatory reaction was dominated by macrophages and giant cells, accompanied by granulocytes and lymphocytes in the early stage; meanwhile, obvious new capillaries and fibroblast proliferation were observed growing inside the implantation areas throughout the entire implantation period. Notably, the trend of inflammatory reaction in the periosteum implantation area was consistent with that in the subcutaneous implantation area, but the overall degree of inflammatory reaction was weaker than that in the subcutaneous implantation area. In addition, compared with the control group, the periosteum of the parietal bone in the implantation area appeared to be slightly thickened (Figure S1).

**Figure 3:**
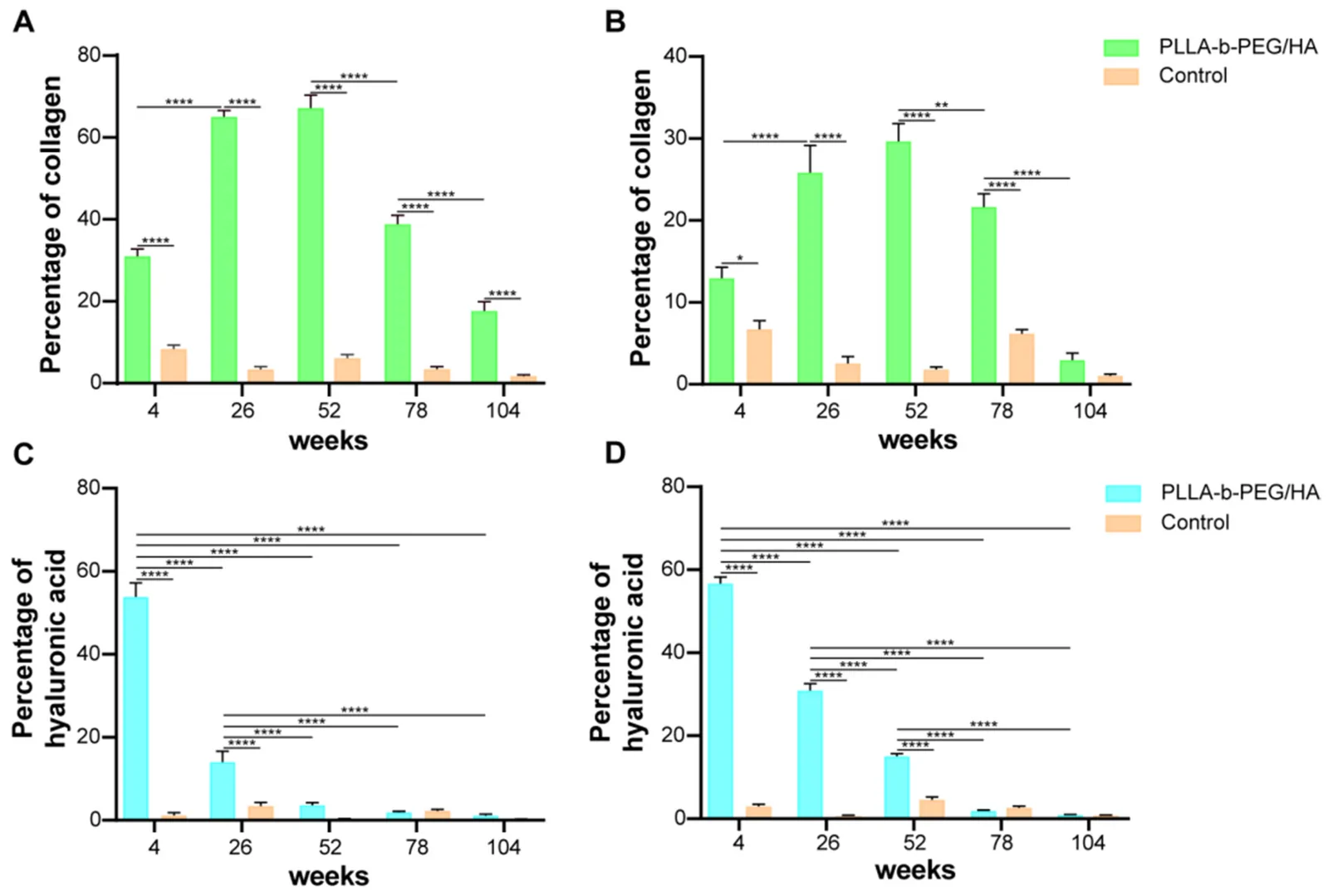
**Dynamic changes in new collagen deposition and hyaluronic acid degradation in distinct tissue sites. New collagen deposition was quantitatively analyzed by Masson staining, while HA degradation was assessed through Alcian blue staining.** (**A**) Dynamic trend of new collagen deposition in the subcutaneous tissue. (**B**) Dynamic trend of new collagen deposition in the periosteum tissue. (**C**) Dynamic trend of HA degradation in the subcutaneous tissue. (**D**) Dynamic trend of HA degradation in the periosteum tissue. * *p* < 0.05, ** *p* < 0.01, **** *p* < 0.0001, n =  6. Data are presented as mean  ±  SEM. SEM, Standard error of the mean.

Masson staining showed that a large number of new blue collagen fibers appeared and gradually increased in the periosteum and subcutaneous implantation areas at 4, 26, and 52 weeks post-implantation, with an orderly arrangement. The ingrowth of new collagen fibers progressed gradually from the outer periphery of the implant toward the inner core, and almost complete collagen ingrowth was observed at 26 weeks post-implantation. More abundant newly formed collagen fibers were distributed around the exposed microspheres, whereas relatively fewer collagen fibers were detected around the microspheres still suspended within the matrix. The number of new collagen fibers began to decrease at 78 weeks and was still slightly higher than that in the control group at 104 weeks. Among them, the new collagen in the periosteum implantation area was looser in morphology than that in the subcutaneous implantation area, and its area ratio was relatively lower at the same time points (Figure 3A,B and Figure S2).

Alcian blue staining revealed obvious blue-stained sodium hyaluronate gel in both the periosteum and subcutaneous implantation areas at 4, 26, and 52 weeks post-implantation. At 4 and 26 weeks post-implantation, the sodium hyaluronate gel gradually degraded, which led to the gradual exposure of PLLA–b–PEG microspheres originally embedded in the gel matrix and sequentially initiated the hydrolytic degradation process of the microspheres. The proportion of sodium hyaluronate gel gradually decreased with prolonged implantation time in both regions; notably, a certain amount of retention was still observable at 52 weeks at the periosteum implantation area, while less residual gel remained in the subcutaneous site at the same time point. This site-specific difference in degradation rate is mainly attributed to weaker tissue reactivity and lower inflammatory cell infiltration in the periosteum region compared with the subcutaneous tissue, which collectively slow HA degradation [18]. At 78 and 104 weeks post-implantation, only hyaluronic acid in the animal’s own extracellular matrix (ECM) was visible in the implantation areas (Figure 3C,D and Figure S3).

Movat’s pentachrome staining indicated that after PLLA–b–PEG/HA filler implantation, newly formed elastic fibers were mainly distributed around collagen tissues proliferating adjacent to microspheres. The quantity of elastic fibers peaked at 26 weeks post-implantation and then gradually declined. Notably, the abundance of elastic fibers in subcutaneous implantation sites was higher than that in periosteal sites at the same time point. Even at 104 weeks after implantation, the elastic fiber content in both regions was still obviously elevated compared with that in blank control tissues (Figure S4).

Herovici’s staining results demonstrated abundant newly synthesized collagen fibers presented as fine thread-like blue-stained structures within the implantation area at 4 and 26 weeks post-implantation. From 52 weeks onward, a large number of mature collagen fibers emerged, which were characterized as thick cord-like structures with pink staining, accompanied by a gradual reduction in nascent collagen. At 104 weeks, the content of newly formed collagen was nearly consistent with that in the blank control group, while the content of thick mature collagen fibers remained distinctly higher than the control level (Figure S5).

Picrosirius red staining showed that the proportion of type III collagen presented a slight upward trend at the time points of 4, 26 and 52 weeks post-implantation (Figure S6).

### 3.4. Scanning Electron Microscopic Observation

**Figure 4:**
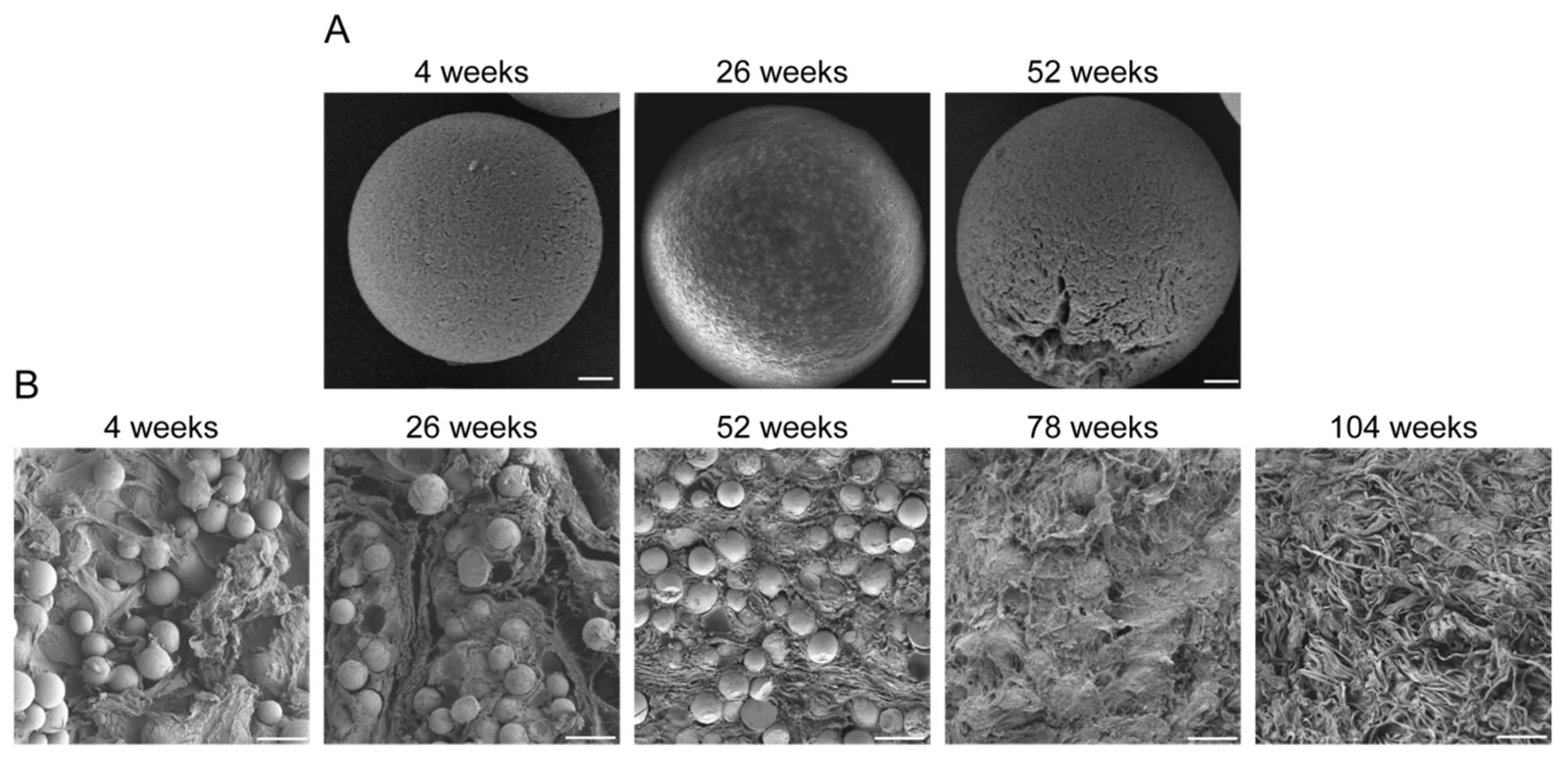
**Degradation characteristics of PLLA–b–PEG microspheres over time, as observed by scanning electron microscopy.** (**A**) In situ morphological features of PLLA–b–PEG microspheres in the subcutaneous tissues at the indicated time points (scale bar, 5 μm). (**B**) Morphology of PLLA–b–PEG microspheres following elution from the host tissue at the indicated time points (scale bar, 50 μm).

SEM observation was conducted to analyze both eluted microspheres and ultrastructural changes of local implanted tissues. In terms of isolated microspheres, intact spherical morphology was still retained at 4 and 26 weeks post-implantation, with initial pores forming on the surface and gradually expanding as implantation time prolonged. Surface defects appeared on microspheres at 52 weeks, and honeycomb-like degraded structures could be seen inside the spheres. Until 78 weeks after implantation, the microspheres were fully fractured and failed to be isolated by elution. For in situ implanted tissues, residual microspheres and sodium hyaluronate matrix were clearly observed at 4 weeks, with only a small number of slender collagen fibers migrating into the filler interior. At 26 weeks, sodium hyaluronate was largely degraded, obvious pores occurred on microspheres, infiltrated collagen fibers increased significantly and mature thick collagen fibers gradually emerged, together with distributed elastic fibers, and the implants were wrapped and separated by collagen bundles. A large number of collagen fibers tightly encased microspheres at 52 weeks, where capillaries and elastic fibers were interspersed among fibrous tissues. Complete spherical microsphere structures were hardly observed at 78 weeks, the implantation area was filled with abundant collagen fibers, accompanied by well-developed blood vessels and elastic fibers, and only a few residual implant fragments could be occasionally found. Up to 104 weeks post-implantation, dense thick mature collagen fibers could still be distinctly detected in the original implantation region (Figure 4).

### 3.5. Molecular Biology Detection

**Figure 5:**
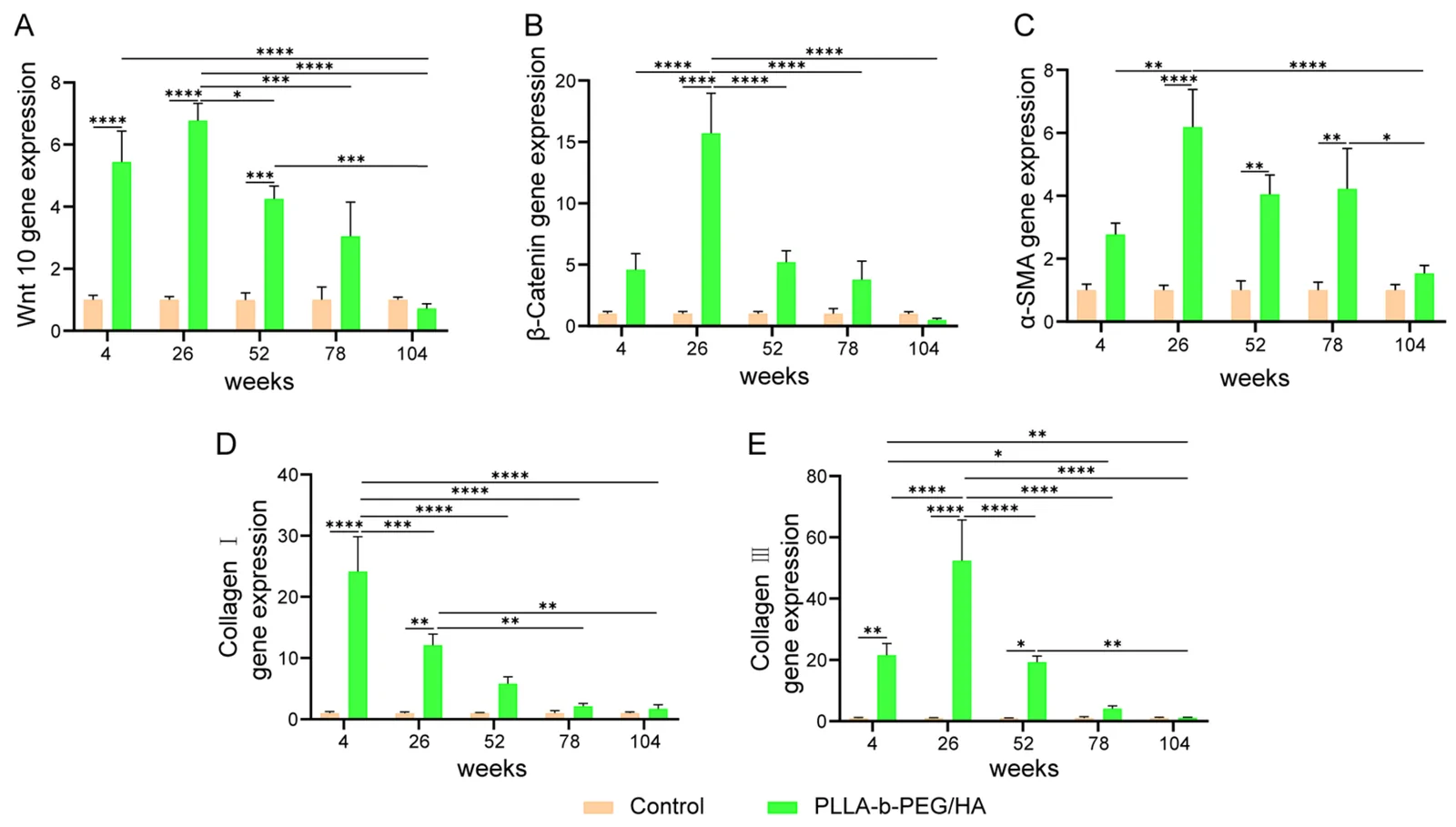
**Detection of gene expression related to Collagen regeneration at different implantation time points in the implantation area by RT-PCR.** (**A**) Gene expression of Wnt10. (**B**) Gene expression of β-catenin. (**C**) Gene expression of α-SMA. (**D**) Gene expression of type I collagen. (**E**) Gene expression of type III collagen. * *p* < 0.05, ** *p* < 0.01, *** *p* < 0.001, **** *p* < 0.0001, n =  6. Data are presented as mean  ±  SEM. Wnt10, Wnt oncogene analog 10; β-catenin, Catenin beta; α-SMA, Alpha-smooth muscle actin; SEM, Standard error of the mean.

To elucidate how PLLA–b–PEG microspheres influence fibroblasts and subsequent collagen production through macrophages, we used RT-PCR to examine the expression changes of collagen synthesis-related genes in the subcutaneous implantation area at different time points after PLLA–b–PEG/HA filler implantation. As shown in Figure 4, the expression levels of WNT10 and β-catenin were both significantly upregulated after filler implantation, which indicates that M1 macrophages began to polarize toward the M2 phenotype at 4 weeks. Additionally, the upregulation of WNT10 occurred earlier than that of β-catenin, which is consistent with the characteristics of the WNT10/β-catenin pathway. As one of the vital signaling pathways regulating elastic fiber formation, the alteration trend of WNT10/β-catenin expression was highly consistent with the dynamic changes of elastic fibers observed via Movat’s pentachrome staining, further confirming that the elevation of elastic fiber content was triggered by the implantation of this composite filler. Accordingly, the upregulation of WNT10/β-catenin signaling-related genes was accompanied by markedly increased α-SMA gene expression after implantation, which is consistent with the extensive fibroblast proliferation observed in H&E staining. Subsequently, the expression levels of Collagen I and Collagen III were significantly elevated, demonstrating active collagen production, which was further confirmed by our Masson staining results. Interestingly, the upregulation of Collagen III gene expression occurred later than that of Collagen I, which is consistent with the observations from Picrosirius red staining (Figure 5).

In addition to macrophages, the expression of inflammatory factors also regulated the tissue response in the implantation area. We detected three major inflammatory factors using enzyme-linked immunosorbent assay (ELISA). We found that the pro-inflammatory factors interleukin-12 (IL-12) and tumor necrosis factor-alpha (TNF-α), as well as the anti-inflammatory factor interleukin-4 (IL-4), all began to increase significantly at 4 weeks post-implantation and started to decrease at 78 weeks post-implantation. Interestingly, IL-12 increased again at 78 weeks post-implantation. We speculate that this finding is associated with the transient increase in macrophages following microsphere fragmentation. The sustained, low-intensity phagocytic stimulus generated by microsphere debris preferentially promotes IL-12 expression, whereas acute-phase cytokines such as TNF-α do not show a secondary surge under such mild persistent stimulation [19]. Meanwhile, significantly elevated anti-inflammatory mediators (e.g., TGF-β) effectively suppress the transcription and secretion of pro-inflammatory cytokines represented by TNF-α [20,21], resulting in this selective cytokine expression profile. This observation is consistent with the high expression of α-SMA at 78 weeks.

**Figure 6:**
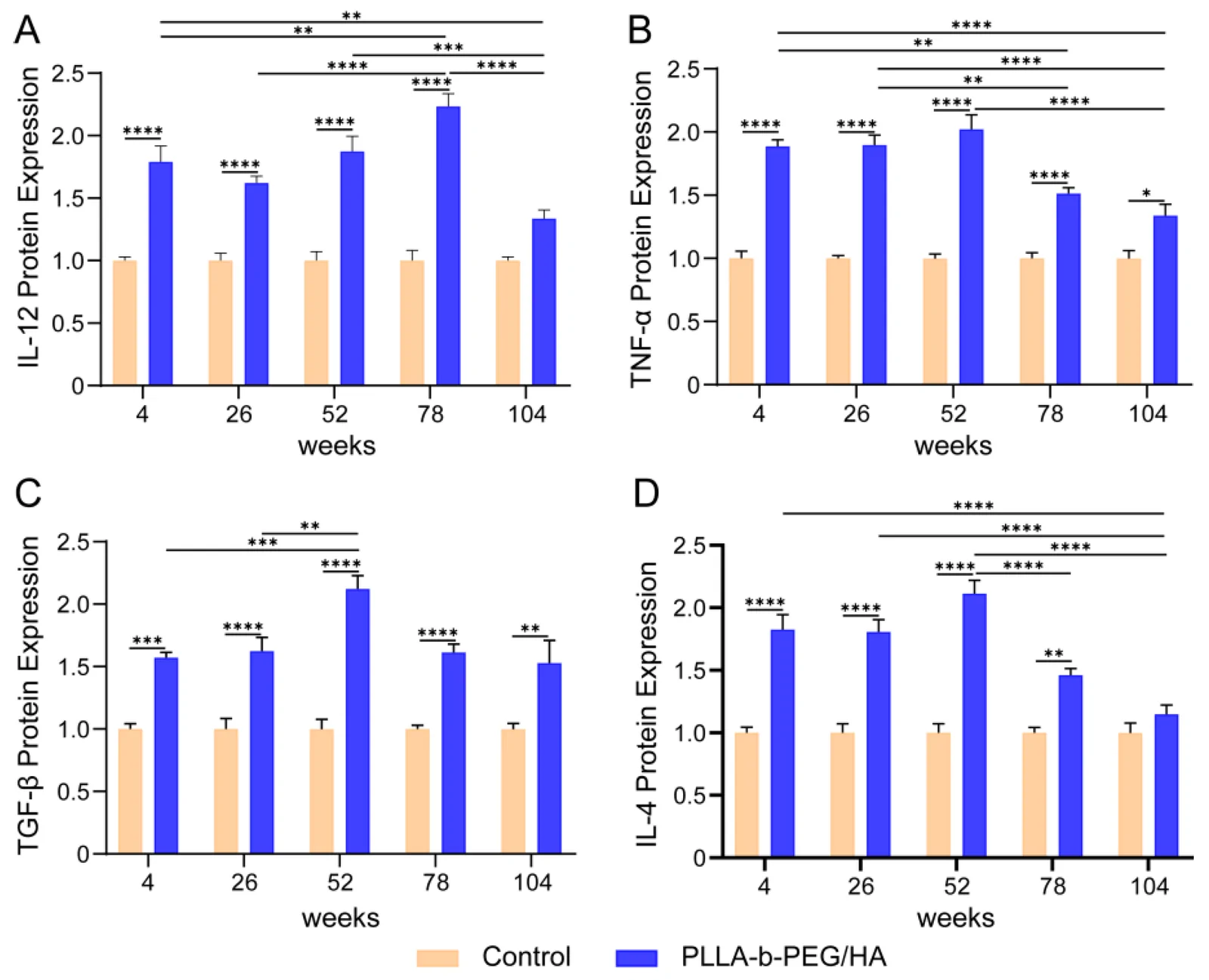
**Detection of protein expression related to inflammatory response at different implantation time points in the implantation area by ELISA.** (**A**) Protein expression of IL-12. (**B**) Protein expression of TNF-α. (**C**) Protein expression of TGF-β. (**D**) Protein expression of IL-4. * *p* < 0.05, ** *p* < 0.01, *** *p* < 0.001, **** *p* < 0.0001, n =  6. Data are presented as mean  ±  SEM. IL-12, Interleukin 12; TNF-α, Tumor necrosis factor-alpha; TGF-β, Transforming growth factor-beta; IL-4, Interleukin 4; SEM, Standard error of the mean.

In addition, we also detected the expression of transforming growth factor-beta (TGF-β), which remained significantly elevated throughout the entire observation period. As a core regulatory protein governing elastic fiber synthesis, its expression trend was highly consistent with the dynamic changes of elastic fibers observed in pathological staining results, which was also in accordance with the sustained synthesis of both collagen fibers and elastic fibers after filler implantation (Figure 6).

## 4. Discussion

This study integrated in vitro laboratory assessment with a 104-week rabbit subcutaneous implantation model to evaluate the long-term degradation profile, tissue remodeling properties and local in vivo biocompatibility of the novel PLLA–b–PEG/HA composite filler. In vitro data confirmed that PEG modification retained the fundamental material characteristics of PLLA while effectively boosting its hydrophilicity and biocompatibility. Long-term in vivo observations further demonstrated that the filler underwent stable and predictable degradation without drastic inflammatory fluctuations. Filler implantation was associated with a balanced local inflammatory microenvironment and upregulated expression of TGF-β and Wnt10/β-catenin signaling molecules, which may contribute to the synchronous regeneration of collagen and elastic fibers. Differential tissue responses across various implantation layers were also identified, solidifying its satisfactory long-term biosafety and reliable tissue remodeling capacity.

The preliminary physicochemical characterizations lay a solid foundation for interpreting the in vivo performance of the composite filler. As comprehensively demonstrated by the in vitro physicochemical characterizations detailed in Section 3.1, SEM, XRD, FTIR, TG and DSC analyses collectively confirmed that PEG modification did not compromise the core physicochemical properties of pristine PLLA. XRD patterns confirmed that PLLA–b–PEG retained the typical α-crystal structure of PLLA with no new crystalline phases formed; FTIR spectra showed identical characteristic functional group peaks, indicating no chemical structural changes occurred during the modification process. TG and DSC results further verified that the thermal decomposition behavior, glass transition temperature and melting point of PLLA–b–PEG were comparable to those of pure PLLA. Notably, SEM observations revealed that PEG-modified microspheres could be uniformly wrapped by lyophilized sodium hyaluronate without aggregation, which directly evidenced the improved hydrophilicity of the material. Taken together, these findings confirm that PEG functionalization effectively enhances the hydrophilicity of PLLA while fully preserving its essential physicochemical characteristics required for biomedical applications.

Traditional PLLA fillers achieve soft tissue augmentation mainly through inherent biostimulatory effects, whereas unregulated foreign body reactions frequently lead to granuloma formation, scar hyperplasia and adverse inflammatory events [22,23]. To address this defect, PEG was applied for surface modification to optimize material hydrophilicity and biocompatibility, and our experimental results validated that such modification improved hydrophilic performance without altering core material properties during fabrication [24,25].

High-frequency ultrasound examinations revealed a statistically significant gradual reduction in filler volume, which is consistent with the inherent biodegradable property of aliphatic polyesters [26]. Distinct from the abrupt fragmentation degradation mode of conventional unmodified PLLA, this modified filler degrades in a regular honeycomb hollow pattern. Conventional PLLA suffers from unstable degradation rates and prolonged in vivo residence time, and sudden rupture of highly crystalline microspheres tends to trigger severe delayed foreign body inflammation [27,28,29]. The steady volume reduction of PLLA–b–PEG/HA is mainly attributed to incorporated hydrophilic PEG segments, which elevate microsphere water absorption and standardize hydrolysis progression, thus avoiding the production of sharp rigid degradation fragments. Additionally, the honeycomb porous structure provides favorable anchoring sites for fibroblast adhesion and growth, guiding inward ingrowth of newly synthesized collagen and stabilizing the filler at implantation sites without displacement. In this manner, the filler can be steadily substituted by autologous functional tissues without inducing acute inflammatory responses.

These favorable biological behaviors were further verified via histopathological analysis. PEG-modified microspheres elicited mild and steady inflammatory responses obviously milder than those induced by unmodified PLLA, which was in line with previous research outcomes [30]. No prominent inflammatory peak appeared in the early implantation phase, and the inflammatory intensity was negatively correlated with the degradation progress of hyaluronic acid. It indicates that the combination of HA and microspheres exerts intrinsic anti-inflammatory activity [31], and HA can also retard lactic acid release during microsphere degradation to alleviate inflammatory aggravation, which has been confirmed by the detection of inflammatory cytokines. Notably, no abnormal inflammatory elevation or excessive fibrosis was observed at week 78, a critical time point when conventional PLLA tends to induce granuloma, proving the biosafety of PEG-modified microspheres even after structural fragmentation.

Robust collagen accumulation was persistently detected at implantation sites throughout the whole experimental period until complete filler degradation. In the early stage, newly formed filamentous immature collagen dominated local tissues along with an elevated proportion of type III collagen. Combined with the regulatory effect of HA [32,33], this tissue composition effectively prevents local tissue sclerosis and promotes the formation of well-organized collagen tissues in the middle and late stages. Apart from potent collagen-promoting effects, this composite filler possesses unique superiority in facilitating elastic fiber regeneration, which is rarely achieved by traditional PLLA fillers that only induce dense and stiff collagen hyperplasia [34,35,36]. The HA matrix establishes a moist and physiological microenvironment in the early post-implantation period, and the PEG-modified microsphere surface reduces non-specific protein adsorption and excessive macrophage adhesion. Such optimized foreign body response inhibits excessive macrophage fusion and foreign body giant cell formation, prevents dense fibrotic scar tissue generation, and promotes the formation of regenerative tissues with evenly distributed collagen and elastic fibers to reconstruct youthful physiological extracellular matrix.

One representative finding of this study is the sustained upregulation of TGF-β expression lasting for 104 weeks, accompanied by increased type III collagen level and continuous neoelastogenesis confirmed by Movat’s pentachrome staining. The sustained collagen-stimulating effect originates from mild subclinical foreign body reactions triggered by PLLA, in which persistent TGF-β signaling drives fibroblast differentiation into myofibroblasts and accelerates extracellular matrix deposition to enhance tissue thickness and skin quality [37,38,39]. TGF-β works synergistically with the Wnt10/β-catenin pathway to co-regulate elastic fiber formation [40,41]. As the filler degrades progressively, autologous functional tissues continuously replace filler volume to maintain long-term structural support. The coordination between IL-4-mediated M2 macrophage polarization [42,43]. and TGF-β-associated tissue remodeling align with the observed tissue repair process and lowers the risk of excessive fibrosis, indicating that the filler may guide host tissues toward physiological regeneration rather than simple fibrotic proliferation, thus supporting high-quality balanced soft tissue remodeling.

Several limitations of this study should be acknowledged. First, this study did not include a head-to-head comparison with conventional unmodified PLLA fillers, which would have provided more direct quantitative evidence for the superiority of PEG modification. Second, only key genes and representative cytokines of the TGF-β/Wnt10/β-catenin signaling pathway were detected; the complete protein expression profile and post-translational modifications of the pathway components were not fully characterized. Third, all experiments were performed in a rabbit subcutaneous implantation model, which differs from human facial soft tissues in anatomical structure, immune response characteristics and metabolic kinetics, thus the extrapolation of these results to clinical applications requires further validation. Nevertheless, these limitations do not compromise the main conclusions of this study, and future investigations will address these issues to provide more comprehensive evidence for the clinical translation of this novel composite filler.

## 5. Conclusions

This study integrated in vitro physicochemical characterization with a 104-week rabbit subcutaneous implantation model to systematically evaluate the long-term biological performance of the novel PLLA–b–PEG/HA composite filler. We demonstrated that PEG functionalization effectively enhances the hydrophilicity of PLLA while fully preserving its core physicochemical properties during fabrication, addressing the inherent limitations of conventional unmodified PLLA fillers. This composite filler exhibits a well-orchestrated dual-action mechanism: the HA matrix provides immediate structural support and volumetric correction, while PLLA–b–PEG microspheres mediate sustained constructive tissue remodeling, enabling synchronous regeneration of type III collagen and elastic fibers. Notably, HA plays a pivotal role in modulating local tissue homeostasis by attenuating early inflammatory responses and constructing a favorable regenerative microenvironment. Collectively, this filler possesses excellent long-term biocompatibility and holds significant clinical potential for natural and durable soft tissue augmentation.

## Data Availability

The original contributions presented in the study are included in the article/Supplementary Material, further inquiries can be directed to the corresponding authors.

## Supplementary Materials

The following supporting information can be downloaded at: https://www.mdpi.com/article/10.3390/jfb17090460/s1, Figure S1: Histological response of periosteum and subcutaneous tissues to PLLA–b–PEG/HA filler injection determined via H&E staining (scale bar, 100 μm). H&E, hematoxylin and eosin; Figure S2: Histological response of periosteum and subcutaneous tissues to PLLA–b–PEG/HA filler injection determined via Masson’s trichrome staining (scale bar, 100 μm); Figure S3: Histological response of periosteum and subcutaneous tissues to PLLA–b–PEG/HA filler injection determined via Alcian blue staining (scale bar, 100 μm); Figure S4: Histological response of periosteum and subcutaneous tissues to PLLA–b–PEG/HA filler injection determined via Movat’s Pentachrome staining (scale bar, 100 μm); Figure S5: Histological response of periosteum and subcutaneous tissues to PLLA–b–PEG/HA filler injection determined via Herovici’s Histology staining (scale bar, 100 μm); Figure S6: Histological response of periosteum and subcutaneous tissues to PLLA–b–PEG/HA filler injection determined via Picrosirius red staining (scale bar, 100 μm).
