# Supplementary material for: Long-Term In Vivo Biological Performance of PLLA–b–PEG/HA Filler
Source: J Funct Biomater. 2026 Sep 8;17(9):460. doi: 10.3390/jfb17090460 (PMC13608104; doi:10.3390/jfb17090460)
Supplement: Supplementary file 1 [file jfb-17-00460-s001.zip › jfb-4451128-supplementary.pdf]

supplementary information

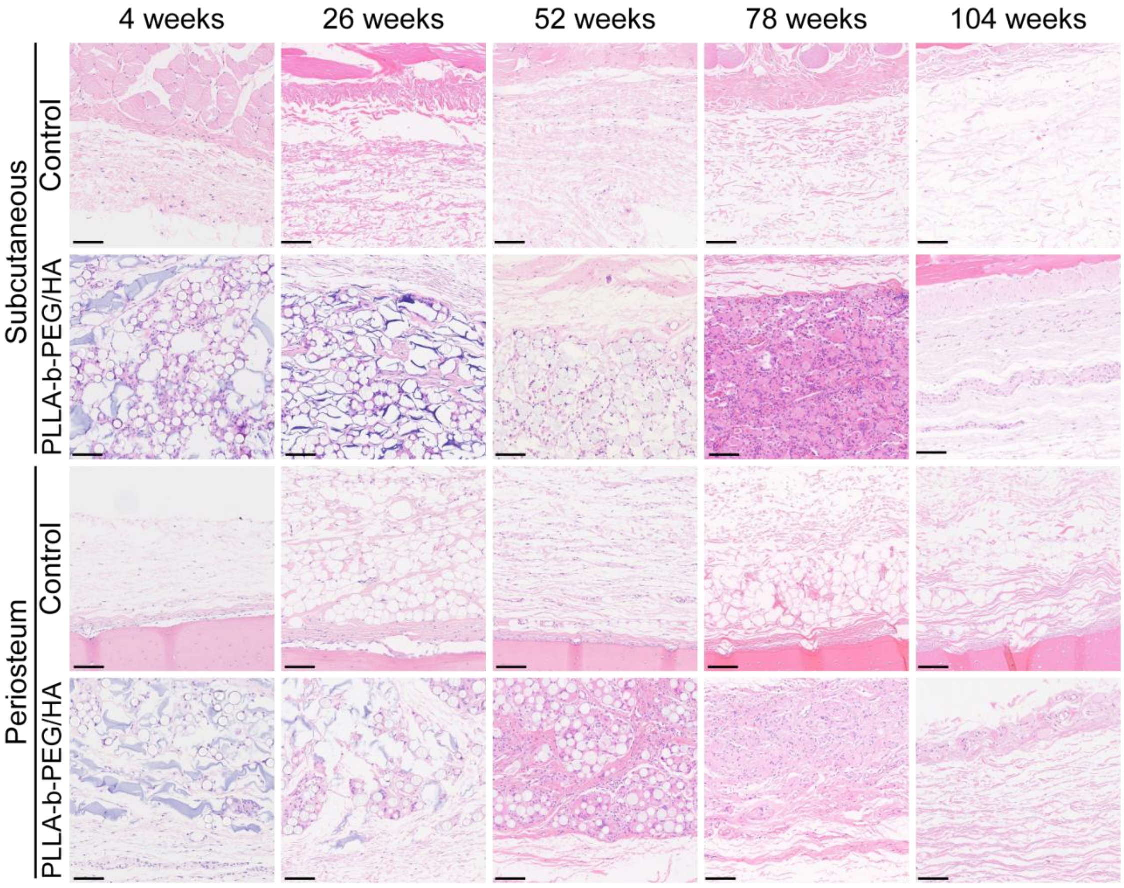

Figure S1: Histological response of periosteum and subcutaneous tissues to PLLA-b-PEG/HA filler injection determined via H&E staining (scale bar, 100 μm).

H&E, hematoxylin and eosin.

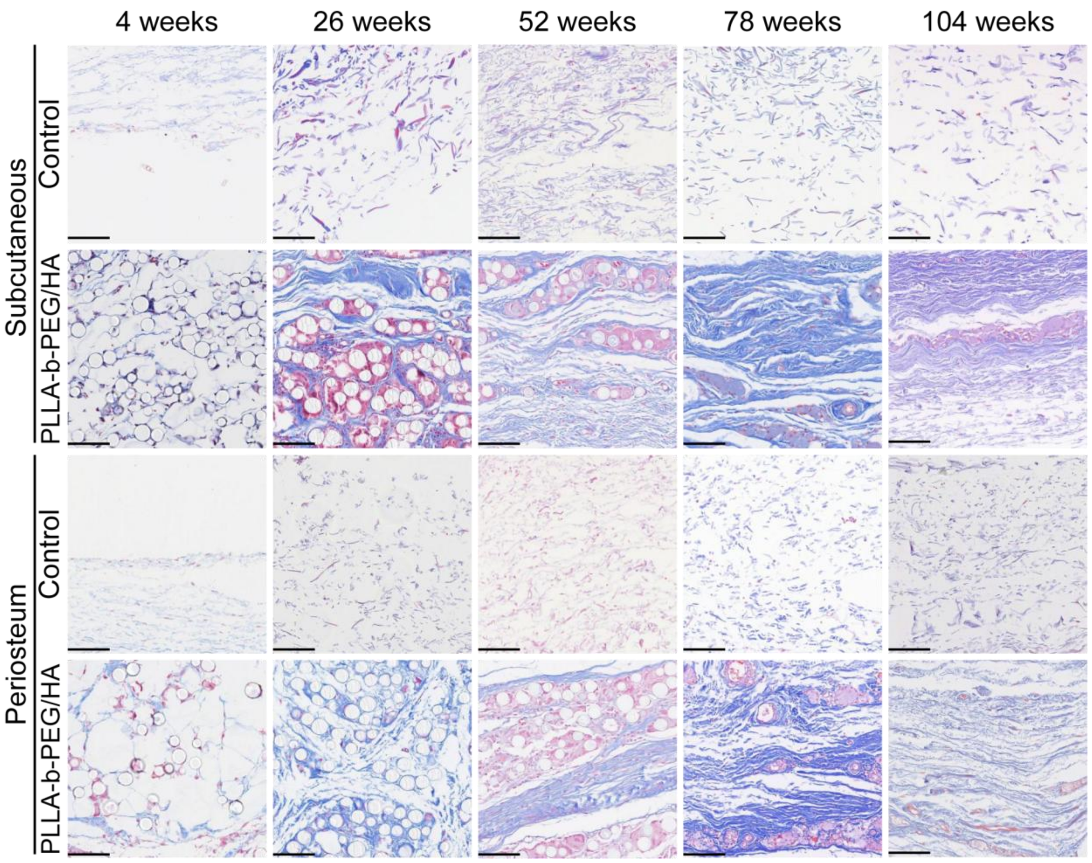

Figure S2: Histological response of periosteum and subcutaneous tissues to PLLA-b-PEG/HA filler injection determined via Masson's trichrome staining (scale bar, 100 μm).

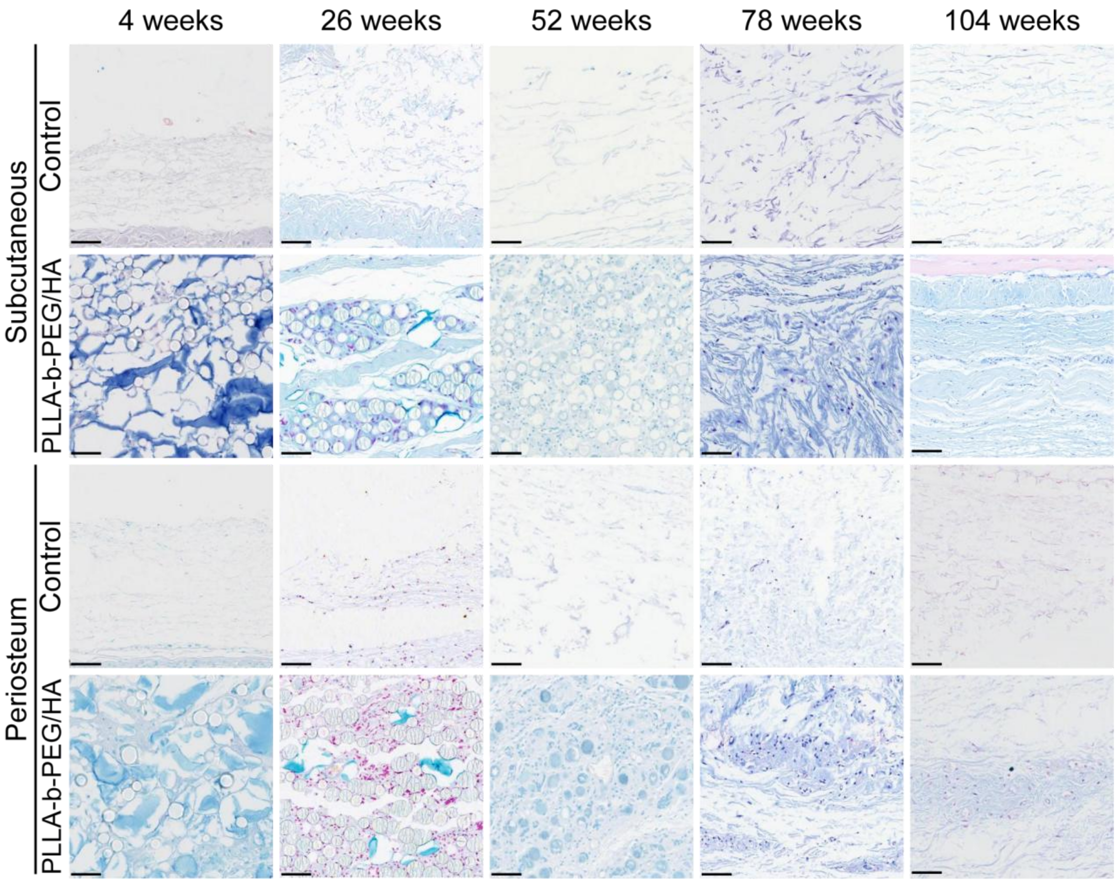

Figure S3: Histological response of periosteum and subcutaneous tissues to PLLA-b-PEG/HA filler injection determined via Alcian blue staining (scale bar, 100 μm).

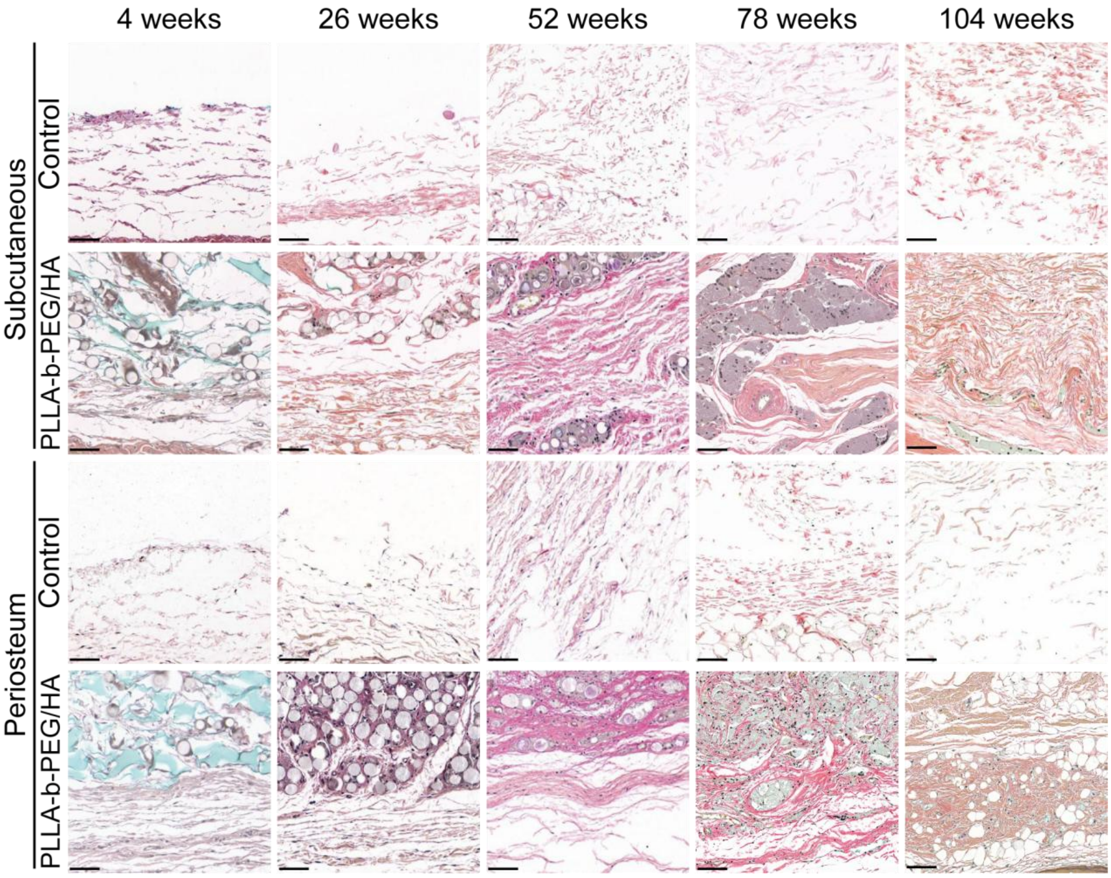

Figure S4: Histological response of periosteum and subcutaneous tissues to PLLA-b-PEG/HA filler injection determined via Movat's Pentachrome staining (scale bar, 100 μm).

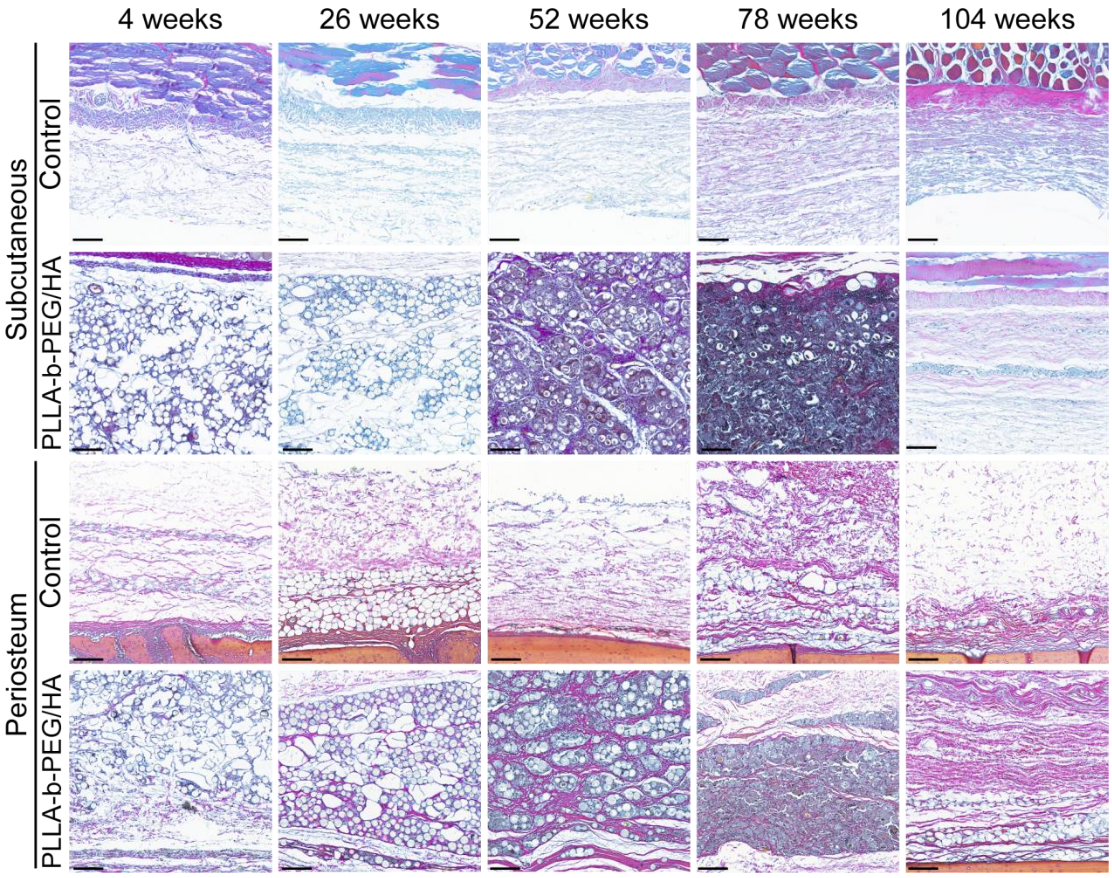

Figure S5: Histological response of periosteum and subcutaneous tissues to PLLA-b-PEG/HA filler injection determined via Herovici's Histology staining (scale bar, 100 μm).

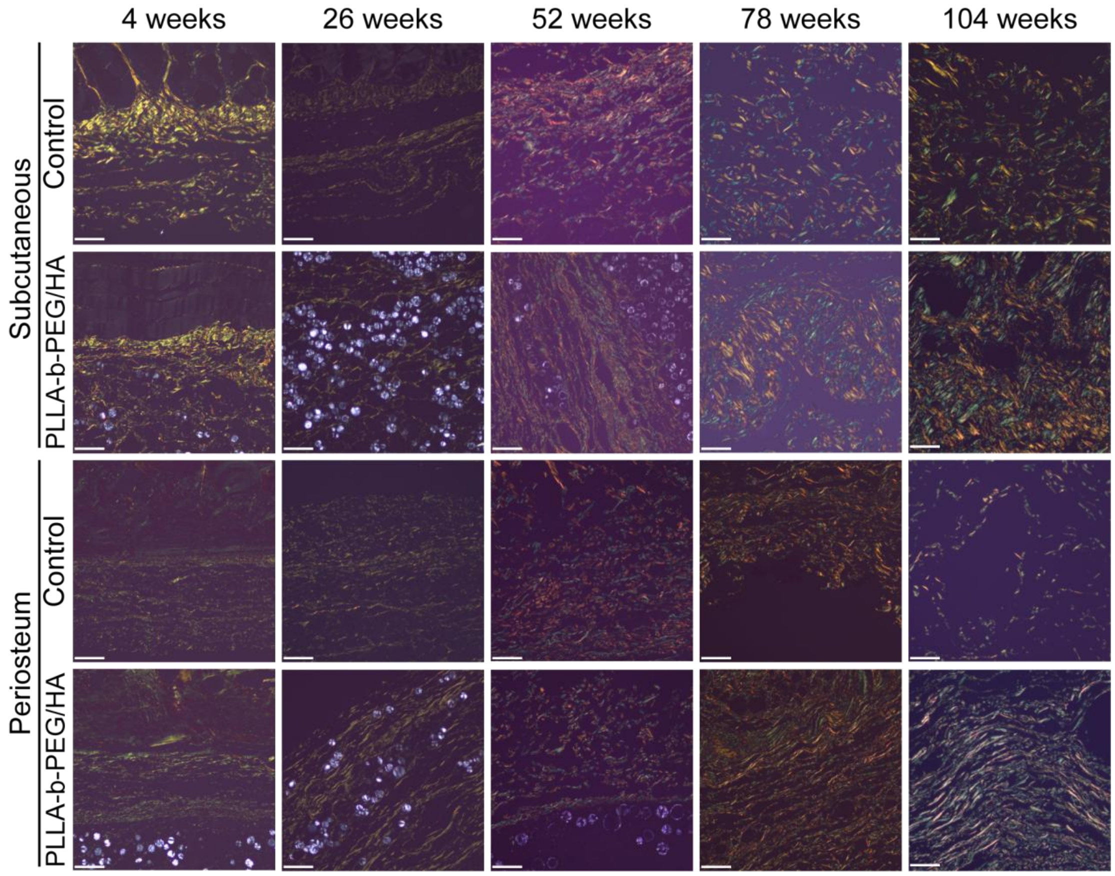

Figure S6: Histological response of periosteum and subcutaneous tissues to PLLA-b-PEG/HA filler injection determined via Picrosirius red staining (scale bar, 100 μm).
